# Personality traits favourable for non-adherence to treatment in patients with chronic myeloid leukaemia: role of type A and D personality

**DOI:** 10.1186/s13030-023-00261-w

**Published:** 2023-01-19

**Authors:** Anna Rychter, Joanna Miniszewska, Joanna Góra-Tybor

**Affiliations:** 1 Department of Haematology, Medical University of Lodz, Lodz, Poland; 2 Department of Health Psychology, Institute of Psychology, Lodz University, Lodz, Poland

**Keywords:** Personality traits, Type A personality, Type D personality, Chronic myeloid leukemia, Adherence

## Abstract

**Background:**

The introduction of BCR-ABL tyrosine kinase inhibitors (TKIs) to chronic myeloid leukemia (CML) therapy has revolutionized the treatment of this disease. Although regular TKI intake is a prerequisite for successful therapy, it has been shown that a significant proportion of patients are non-compliant. Recently there is growing evidence that personality traits may influenced the tendency for non-adherence to treatment in patients with chronic diseases. As far as we know, such a relationship in patients with CML has not been examined, yet. The aim of our study was to determine if personality traits favor non-adherence to treatment recommendations. We investigated the relationship between five-factor model personality factors (conscientiousness, neuroticism, agreeableness, extraversion, and openness) and medication non-adherence. We also checked if the patients with type A and type D personality, were at higher risk of poor medication adherence.

**Methods:**

The following tools were used: self-constructed survey, the NEO-Five Factor Inventory, the Framingham Type A Scale, the D-Scale 14. The study included 140 CML patients treated with imatinib, dasatinib, or nilotinib.

**Results:**

39% of patients reported skipping at least one dose of medication in the month prior to follow-up visit. 51% admitted to skipping such doses from the start of their treatment to the time at which our assessment was performed. We did not find any relationship between the mean values of the analyzed factors of the Big Five (neuroticism, extraversion, openness, agreeableness, conscientiousness) and adherence. However, our analysis revealed that CML patients who admitted to missing doses of drugs during the entire course of treatment demonstrated greater intensity of type A personality traits (*p* = 0.020). Regarding both factors of type D personality, it was revealed that higher level of negative affectivity significantly decreased the adherence (*p* = 0.020).

**Conclusion:**

The results of our study indicate that screening for type D and A personalities may help to identify patients who are at higher risk of poor medication adherence.

## Background

Non-adherence is a common problem in patients with chronic diseases receiving oral medications. With the spread in oral anti-cancer therapies, the problem of low ‘adherence’ also increasingly applies to cancer patients [1–5].

Chronic myeloid leukaemia (CML) is myeloproliferative neoplasm characterized by the presence of the Philadelphia (Ph) chromosome and *BCR-ABL* gene, which encodes for a protein with deregulated tyrosine kinase (TK) activity, crucial for malignant transformation in CML. Deregulated Bcr-Abl TK activity is the target for the new, very effective agents introduced to first line CML therapy – BCR-ABL TK inhibitors (TKIs): imatinib, dasatinib, and nilotinib. Introducing TKI therapy for CML has changed the treatment paradigm for this disease and extended survival to a period comparable to a healthy population. All TKIs are oral medications, taken on one’s own by the patient. Among many factors influencing an optimal effect of treatment, proper patient adherence is one of the most important. Non-adherence to TKI treatment may have very serious implications such as lack of response, CML progression, and death. Therefore, identification of factors influencing compliance may be essential to successful treatment.

Many studies have attempted to identify factors associated with increased nonadherence among CML patients [1, 3–5]. There are conflicting data regarding the influence of age, gender, education, comorbidities, and adverse events on the level of adherence [1, 4–7]. Our previous study revealed that adherence deteriorates over time, furthermore, patients aged > 65 years and patients suffering at least one comorbid disease had better adherence [7].

Recently there is growing evidence that personality traits may influenced the tendency for non-adherence to treatment in patients with chronic diseases [8–11].

One model that is commonly used to describe personality is the five-factor model (FFM) of personality, also known as Big Five, which describes personality according to traits: Neuroticism, Extraversion, Openness to experiences, Agreeableness, and Conscientiousness. There are papers which revealed that neuroticism negatively influenced adherence, whereas individuals scoring high on agreeableness and conscientiousness tended to be more adherent to treatment [8–11]. Type A refers to a pattern of behavior and personality associated with high achievement, competitiveness, and impatience. However, due to strong opinions that this personality type is treated too generally, it was started to be perceived as a lifestyle. Most of the studies conducted so far have focused on personality type A and its association with coronary artery disease [12–16], although not all researchers confirmed the existence of this relationship. Certain aspects of this personality type - not expressing negative feelings, especially suppressing hostility - have been recognized as being a significant cause of ischemic heart disease. It was recognized that they may be more unfavorable to health than other components and may increase the risk of various other diseases, not only ischemic heart disease as previously thought [17, 18]. Various researchers tend to believe that personality type A may also contribute to the development of other somatic diseases such as gastric and duodenal ulcers, digestive system diseases, and rheumatic disease [15, 19]. Later research revealed that it is not so much the type A personality that is conducive to getting sick, but its 2 basic elements, i.e. anger and hostility [20]. Not only the occurrence of type A personality turned out to be significant, but also its connection with the way of coping with emotions [21]. The researchers’ reservation ns also concerned the too general treatment of this type of personality: in the original sense it encompassed both personality traits and behavioral elements, which predisposed the perception of this behavior pattern more as a lifestyle than a personality type. Further research on negative feelings (anger) and hostility did not provide a clear answer to the questions about their role in the development and progression of diseases [22]. To the best of our knowledge, there are no studies that show a relationship between personality type A and medical adherence in patients with CML. In the 1990s Johan Denollet introduced the idea of type D personality, which is a distressed personality that includes two stable personality traits: negative affectivity (NA) and social inhibition (SI). Many investigators have investigated the association of type D personality with clinical outcomes. Type D has been linked to poor adherence to treatment regime in sleep apnea patients [23], patients with heart failure [24], myocardial infarction [25], and acute coronary syndrome [26].

As far as we know, such a relationship in patients with CML has not been examined, yet.

## Aim of the study

The aim of the study was to identify psychological factors possibly related to non-adherence to therapeutic recommendations in patients with CML. We investigated the relationship between five-factor model personality factors (conscientiousness, neuroticism, agreeableness, extraversion, and openness) and medication non-adherence. We also checked if the patients with type A and type D personality were at higher risk of poor medication adherence.

## Patients and methods

Detailed eligibility criteria and methods of medication adherence assessment have been published elsewhere [7]. Briefly, the inclusion criteria to the analysis were as follows: (1) age ≥ 18 years; (2) diagnosis of chronic phase CML; (3) imatinib, dasatinib, or nilotinib treatment for at least 6 months. The study was conducted anonymously and voluntarily. All patients participating in the study provided written informed consent.

The exclusion criterion was an intellectual handicap which prevented the participant from understanding questions.

The basic study tools were questionnaires based on the diagnostic survey method and consisted of 17 closed questions which the patients replied to at their follow-up doctor’s appointments designed to evaluate non-adherence levels in taking medication. Questions included those on how many doses were skipped within the last month prior to the visit as well as over the entire treatment period.

The NEO-Five Factor Inventory, the Framingham Type A Scale, and the D-Scale 14 were used to assess the personality traits [27–33].

- The NEO-Five Factor Inventory is based on a five-factor personality model, called the “Big Five”. The inventory is a sixty-item questionnaire, and the respondent’s answers are evaluated according to a five-point Likert’s Scale. The scores are gathered into 5 scales related to neuroticism, extroversion, openness to experience, agreeableness, and conscientiousness [27, 28].
- The Framingham Scale is used for assessment the intensity of type A [31]. It consists of 10 statements. The first five refer to traits and characteristics typical for an individual; another four to feelings observed at the end of a normal day, and the last time pressure [29–31].
- The D Scale was created to measure type D personality [32, 33]. The scale consists of 14 statements, 7 of which refer to negative affectivity and the remaining seven to social inhibition. Negative affectivity is manifested by a tendency to experience negative emotions (anger, fear, irritability, or depression), whereas social inhibition is identified by avoidance of expressing these negative emotions and behaviour triggered by them.

## Ethical issues

The study was approved by the Bioethics Committee of the Medical University of Lodz (No. RNN/226/15/KE).

## Statistical analysis

The authors applied the t-Student test and one-way analysis of variance (ANOVA). A value of 0.05 was adopted as statistically significant. The Statistica 10.0 MR 1 programme was used for statistical purposes.

## Results

The study included 140 patients with a median age of 58 years (range 22-85 years). There were 70 men (50%) and 70 women’s (50%). Imatinib was administered to 101 patients, 25 received dasatinib, and 14 were given nilotinib. Of the 140 subjects, two did not reply as to whether they skipped medication doses in the month prior to the doctor’s appointment. Of the remaining138 subjects, 54 respondents (39%) reported skipping at least one dose of medication in the month prior to follow-up visit. Of the 140 subjects, 72 (51.4%) admitted to skipping such doses from the start of their treatment to the time at which our assessment was performed.

The five-factor personality model found that female subjects demonstrated a higher level of neuroticism (*p* = 0.001) and agreeableness (*p* = 0.023) than did males. We did not find any relationship between the mean values of the analysed factors of the Big Five (neuroticism, extraversion, openness, agreeableness, conscientiousness) and adherence (Table 1).

**Table 1:** Univariant analysis of the influence of NEO-FFI personality dimensions on skipping TKI doses during the entire treatment period in a group of chronic myeloid leukemia patients (*n* = 140)

Variable	Patients who reported dose skipping (*n* = 72)		Patients who did not report dose skipping (*n* = 68)		t	df	*P *value
	M	SD	M	SD			
**NEO-FFI Neuroticism**	19.4	7.4	17.9	6.7	1.28	138	0.201
**NEO-FFI Extraversion**	28.5	7.2	28.3	6.4	0.14	138	0.891
**NEO-FFI ** **Openess**	24.9	6.4	24.3	6.0	0.56	138	0.580
**NEO-FFI Agreeableness**	32.8	6.7	33.5	5.2	-0.73	138	0.466
**NEO-FFI Conscientiousness**	36.4	6.7	36.1	5.8	0.29	138	0.774

*M *Mean, *SD *Standard deviation, *df *Degrees of freedom

However, our analysis revealed that CML patients who admitted to missing doses of drugs during the entire course of treatment demonstrated greater intensity of type A personality traits (*p* = 0.020). Regarding both factors of type D personality, it was revealed that a higher level of negative affectivity significantly decreased the adherence (*p* = 0.020). The results are presented in Table 2.

**Table 2:** Univariant analysis of the influence of personality dimensions A and D, on skipping TKI doses throughout the treatment period in a group of chronic myeloid leukemia patients CML (*n* = 140)

Variable	Patients who reported dose skipping (*n* = 72)		Patients who did not report dose skipping (*n* = 68)		t	df	*P *value
	M	SD	M	SD			
**Typ A**	5.3	1.9	4.6	1.8	2.36	138	**0.020**
**Typ D:**							
** DS14 ** **Negative Affectivity**	12.9	6.4	10.6	5.1	2.35	138	**0.020**
** DS14 ** **Social Inhibition**	9.7	5.9	9.3	5.3	0.39	138	0.700

*M *Mean, *SD *Standard deviation, *df *Degrees of freedom

Although our analysis of type D confirmed that female subjects demonstrated an elevated level of negative affectivity as compared to males (*p* = 0.017) we did not show reduced compliance among women.

## Discussion and conclusion

As far as we know, this study is the first to analyse the relationship between NEO-FFI personality dimensions, type A nad D personality and the level of adherence in CML patietnts.

Our study revealed that a significant proportion of the surveyed CML patients treated with TKI did not adhere to treatment recommendations. Half of the patients admitted to skipping at least one drug dose during the entire course of treatment and 39% did so within their last treatment month.

Regarding the influence of personality on compliance, we demonstrated that patients with CML who admitted to missing doses of drugs had greater intensity of type A personality traits. Type A personality, which identifies an anxious, hyperactive, and hostile subject, has an impact on patients’ way of health control. Several studies have emphasized a significant contribution of type A personality to development of coronary artery disease [12–16]. A study by Miličić et al. revealed that patients with type A personality hospitalized due to acute myocardial infarction tended to demonstrate behaviours which favour development and occurrence of complications of coronary disease [34]. Similar to the observations of Miličić et al., our analysis has shown that type A personality negatively affects adherence. Type A people had a tendency to miss drug doses. Patients with type A personality are characterized by strong fear, which undoubtedly has an impact on their way of health control. However, it should be taken into account that the observations of Miličić et al. were related to pro-health behaviors (cardiac rehabilitation) rather than regularity of drug dosing. Our study is most likely the first to attempt such an assessment, thus we were unable to find articles that we could refer to.

We also demonstrated that patients with type D personality had worse compliance to TKI treatment. Type D is a distressed personality that includes two stable personality traits: negative affectivity and social inhibition. Many investigators have studied the association of type D personality with clinical outcomes. Type D has been linked to poor adherence to treatment regime in sleep apnea patients [23], patients with heart failure [24], myocardial infarction [25], and acute coronary syndrome [26]. Li et al. [35] obtained similar results in their study on the effect of type D personality on the level of adherence in patients with type 2 diabetes. This personality type was a strong prognosticating factor for non-adherence to medical recommendations. High levels of negative affectivity and social inhibition were prognosticating factors for a low level of adherence. De Fruyt and Denollet [36] confirmed a strong relationship between type D personality traits and two Big Five factors, neuroticism and introversion. Both negative affectivity and neuroticism are manifested by strong susceptibility to stress, a strong feeling of fear and stress. Social inhibition is similar to introversion. However, we did not find any relationship between the mean values of the analysed factors of the Big Five (neuroticism, extraversion, openness, agreeableness, conscientiousness) and adherence. In contrast to our results, Axelsson et al. in a study of a group of 180 patients with asthma and allergic rhinitis confirmed that patients characterized by strong neuroticism were less prone to follow prescribed therapy [9]. Similar observations were made by Bruce et al. [8] in patients with multiple sclerosis: a high level of neuroticism was accompanied by poorer adherence to therapeutic recommendations. Also, Jerant et al. [10] revealed that neuroticism had a negative impact on adherence levels. Regarding the other factors of the Big Five, Skinner et al., in their study, conducted on 1,313 patients with type 2 diabetes [11], observed that people demonstrating higher levels of conscientiousness much more easily followed therapy and those with high levels of openness demonstrated lower BMI and were more prone to have blood glucose tested. In turn, patients with high levels of agreeableness were less prone to adhere to therapeutic recommendations. In contrast Bruce et al. [8] demonstrated that increased openness to experience has a negative impact on adherence levels, whereas conscientiousness has a positive effect on them.

The lack of relationships between personality traits (Big Five) and compliance of CML patients with medical recommendations in our study may result from several aspects. First, the diagnosis of CML as a life-threatening disease can trigger a range of psychological defense mechanisms to manage anxiety. These mechanisms may hinder the rational assessment of the situation and thus prevent adaptive behavior. In the case of chronic diseases that do not directly threaten health, the use of mechanisms may be much less frequent. Features such as agreeableness or conscientiousness may play a significant role in long-term activities, requiring the implementation of various recommendations with varying frequency.Wouters et al. have shown that conscientiousness was associated with better self-reported adherence in patients taking antidepressants. However, there was no association between personality characteristics and adherence as assessed by electronic drug monitoring or dispensation data [37]. Cohen et al. stated that agreeableness was a significant positive predictor of compliance with antidepressant medication [38]. Also Axelsson et al. proved that agreeableness and conscientiousness were positively related to adherence [39].

In another study Adachi et al. showed a link between conscientiousness and adherence.The correlation between personality traits and adherence to medical recommendations in the secondary prevention of cardiovascular disease was investigated. Data from 128 patients were analyzed. Higher conscientiousness scores were significantly associated with high compliance scores. However other combinations of personality traits and medical adherence did not show statistically significant correlations in multivariate analyses [40].

A few studies have shown that extroversion and neuroticism are related to low level compliance or adherence [38, 39, 41].

The cited studies show that adherence has been tested with different tools in various diseases and analyzes and, above all, it does not mean the same (it may only be taking medication, it may be diet, blood pressure measurements, physical activity, etc.). Not all of them showed a relationship between the Big Five and adherence, which is the basis for further studies also involving patients with CML.

It was also checked to see if patients taking IKT differed in the level of the studied variables depending on gender. Women had a higher level of neuroticism and agreeableness than men. Such differences, albeit insignificant, were shown by many other reports, e.g. Feingold [42].

## Limitation

One limitation of the study is the relatively small number of patients, especially in the dasatinib and nilotinib treatment groups. Moreover, the people who volunteered for the study simply might have been more willing to cooperate with a doctor.

A significant limitation of the presented analyzes is the lack of use of a standardized method to assess adherence to recommendations. The respondents answered the questions included in a proprietary questionnaire. Therefore, it is difficult to relate the obtained results to other reports in the literature on the subject. There was also no objective measurement of compliance with them (e.g. concentration of the drug). In future analyzes, it would also be worth monitoring the mental state of patients (e.g. the presence of depression or anxiety disorders), which are common in chronically ill patients. Mental disorders can significantly affect the cognitive abilities of the respondents and thus the way in which they self-evaluate their functioning. It would be also worthwhile to conduct longitudinal analyses (e.g. with triplicate measurements) that give more reliable results.

To conclude: Screening for type D and A personalities may help to identify those who are at higher risk of poor medication adherence. Interventions aiming to improving/enhancing medication adherence need to consider patients with a negative affectivity personality who are at higher risk for medication nonadherence, which may lead to adverse health outcomes. Proper identification of a group of patients particularly prone to non-adherence may help with the introduction of interventions to prevent this phenomenon.

## Data Availability

The datasets generated during and/or analysed during the current study are available from the corresponding author on reasonable request.

## Abbreviations

TKI: Tyrosine kinase inhibitors
CML: Chronic myeloid leukemia
Ph: The Philadelphia chromosome
FFM: Five-factor model of personality, also known as Big Five
NA: Negative affectivity
SI: Social inhibition
BMI: Body Mass Index
NEO-FFI: NEO Personality Inventory.
